# Defect-induced ferromagnetism in a *S* = 1/2 quasi-one-dimensional Heisenberg antiferromagnetic chain compound

**DOI:** 10.1038/s41598-021-93930-1

**Published:** 2021-07-14

**Authors:** Zhe Wang, Lin Hu, Langsheng Lin, Yuyan Han, Ning Hao, Jingtao Xu, Qianwang Chen, Zhe Qu

**Affiliations:** 1 Anhui Key Laboratory of Condensed Matter Physics at Extreme Conditions, High Magnetic Field Laboratory, Hefei Institutes of Physical Sciences, Chinese Academy of Sciences, Hefei, 230031 Anhui China; 2 Science Island Branch of Graduate School, University of Science and Technology of China, Hefei, 230026 Anhui China; 3 CAS Key Lab of Photovoltaic and Energy Conservation Materials, Hefei Institutes of Physical Sciences, Chinese Academy of Sciences, Hefei, 230031 Anhui China; 4 Ningbo Ruiling Advanced Energy Materials Institute Co., Ltd, Ningbo, 315500 Zhejiang China; 5 Hefei National Laboratory for Physical Sciences at Microscale, Department of Materials Science & Engineering, University of Science and Technology of China, Hefei, 230026 Anhui China

**Keywords:** Magnetic properties and materials, Ferromagnetism

## Abstract

We present evidences that defects in the spin *S* = 1/2 Heisenberg antiferromagnetic chain (HAFC) compound can lead to ferromagnetism by studying the magnetic and thermal properties of the newly discovered quasi-one-dimensional (1D) metal–organic framework [CH_3_NH_3_][Cu(HCOO)_3_] (MACuF). Our findings suggest that the long-range ferromagnetic order at 3.7 K can be attributed to Cu^2+^ ions from the 2D networks constructed by the endpoints of the broken chains. In such a case, the intrinsic magnetism can emerge in this quasi-1D Heisenberg chain system at the background of the short-range antiferromagnetism. This unusual ferromagnetism found in HAFC not only enriches magnetic features in the low-dimensional systems, but helps to understand some of the exotic magnetic phenomena in other real quasi-1D magnetic materials.

## Introduction

Mermin–Wagner theorem^1^ indicates that no magnetic long-range order survives due to the strong spin fluctuations until *T* = 0 K in one-dimensional (1D) and two-dimensional (2D) spin systems, regardless of the strength of the exchange interactions between the neighbour spins. While the real materials are three-dimensional by nature, the quasi-1D magnetic systems, where the magnetic interactions proceed predominantly along one direction, provide the platform to verify the predications of the Mermin–Wagner theorem and to unravel new properties beyond the theorem. Many exotic magnetic phenomena, such as Luttinger liquid state^2,3^, continuous incommensurate magnetic phases^4^, topological quantum phase transition^5^, have been experimentally claimed in the quasi-1D Ising chain compounds.

**Figure 1:**
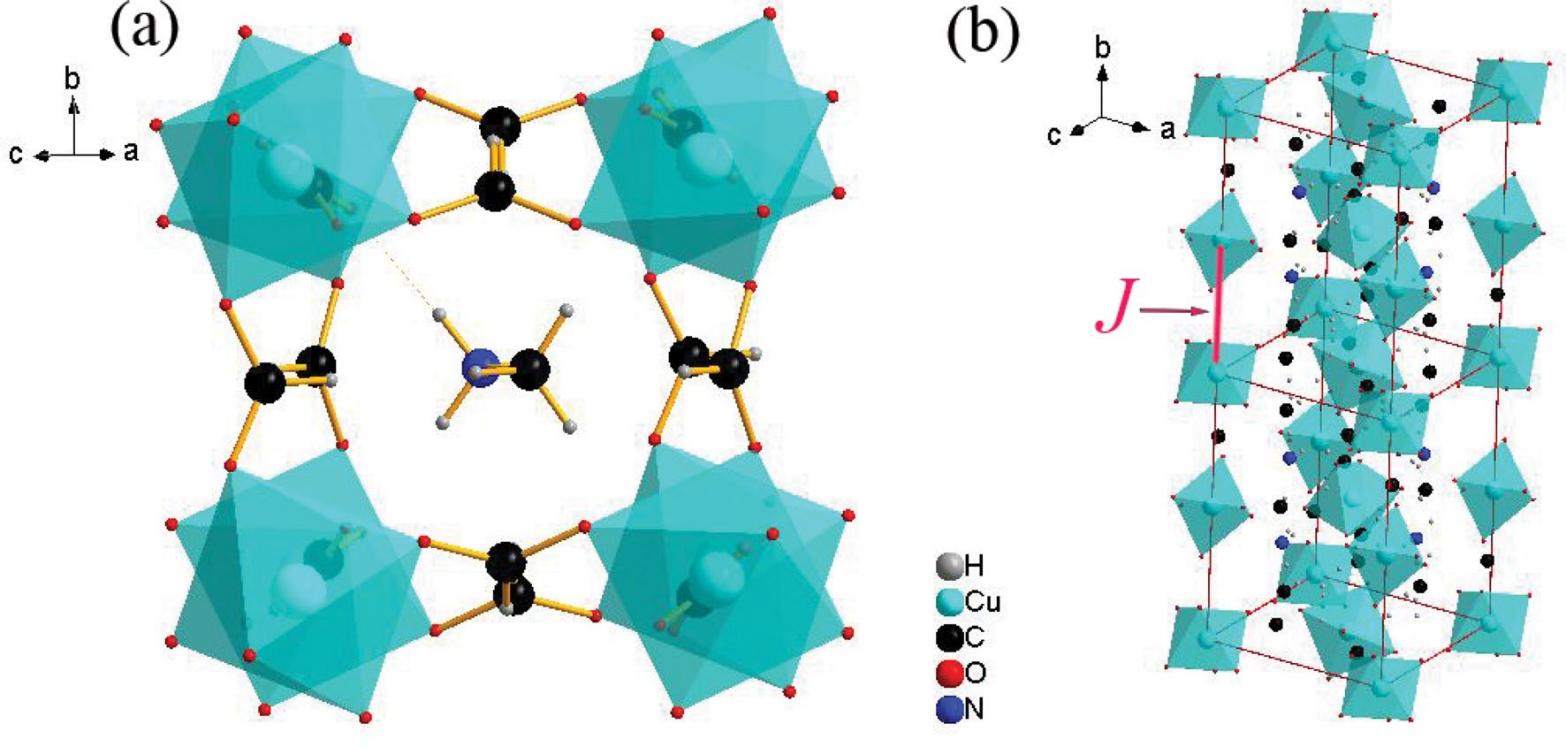
Top view (**a**) and side view (**b**) of the perovskite-like structure of [CH_3_NH_3_][Cu(HCOO)_3_]. CH_3_NH_3_^+^ occupied the pseudo cubic cavity formed by Cu^2+^ and CHOO^−^. Cu^2+^ ions in the endpoints of chains constitute a 2D square lattice. The crystal structure diagram was drew by Diamond (version 4.6.5). http://www.crystalimpact.com/diamond^27^.

Metal–organic framework (MOF) has received much attention in recent years due to its novel optical, magnetic and dielectric properties^6^. It is a kind of crystal network formed by connecting metal ions with organic ligands. Because of the larger size of organic ligands, magnetic elements can be separated efficiently into chains or plains, possessing great advantages in the construction of low-dimensional magnetic materials. In this work, we focus on [CH_3_NH_3_][Cu(HCOO)_3_] (MACuF), a MOF that displays a distorted perovskite-like structure, and has orthorhombic symmetry of space group *Pnma* with *a* = 8.4573 Å, *b* = 11.4362 Å, *c* = 8.1022 Å and *Z* = 4^7^. The structure of MACuF is shown in Fig. 1. The Cu^2+^ ion connects its six nearest HCOO^−^ anions and forms a Cu–O octahedron structure. Due to a strong John–Teller effect, the Cu–O octahedron is strongly distorted, four Cu–O bonds are compressed and the other two Cu–O bonds are elongated, forming a Cu–O–C–O–Cu chain along b axis, which mainly account for the low-dimensional magnetism in this material. The adjacent octahedrons are not coplanar or co-edge, but isolated. Therefore, the magnetic chain formed by this structure is more likely to be strongly isotropic Heisenberg chain. Based on such considerations, B. Pato-Doldán et al*.* considered the anomaly of susceptibility at around 4 K as a sign of the occurrence of long-range AFM phase transition^7^.

In this work, we carry out detailed measurements on magnetic properties and specific heat of MACuF single crystals. The experimental and fitting results prove that this compound is indeed an *S* = 1/2 uniform HAFC system. What's surprising is that the magnetic phase transition at 3.7 K is actually related to a ferromagnetic ordered state. Moreover, the ferromagnetic saturation magnetic moment is almost proportional to the concentration of defects in the paramagnetic state. We suggest that this ferromagnetism in HAFC is attributed to the long-range ferromagnetic order formed by the Cu^2+^ ions in the 2D networks which is formed by chains' defect points. Our work enriches understanding of magnetic features in this interesting material. Magnetism from similar mechanisms may be prevailing in low dimensional materials.

## Results and discussions

**Figure 2:**
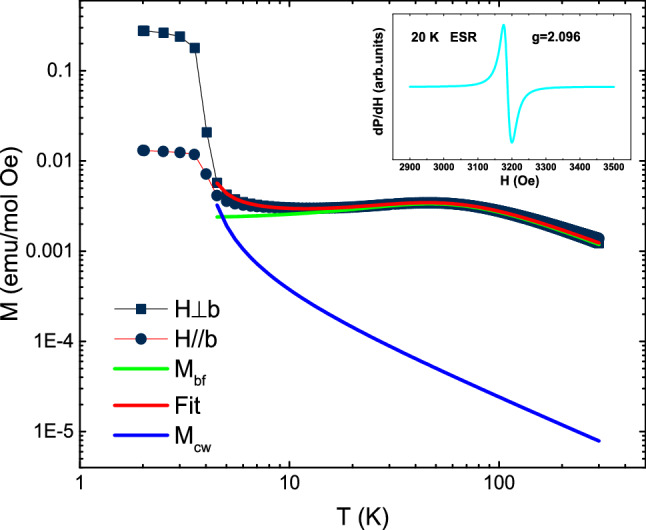
Temperature dependence of the static magnetic susceptibility *χ* = *M*/*H* measured at 200 Oe for *H* parallel and vertical to b axis above 4 K. Static magnetic susceptibility *χ* = *M*/*H* of MACuF, at *B* = 200 Oe. The red line represents the fit by Eq. (2). The green line is contribution of *χ*_chain_. The blue line is Curie–Weiss paramagnetic susceptibility. The inset shows the ESR spectra at 20 K.

Figure 2 presents the temperature dependence of the static magnetic susceptibility *χ* = *M/H* for both *H* parallel and vertical to *b* axis at 200 Oe. A broad maximum around 46 K displays the 1D magnetic nature and indicates a short-range antiferromagnetic exchange interaction^8^. As the temperature decreases, a sudden upturn below 5 K was observed. The susceptibility in both directions is almost identical above 4 K, but the value of susceptibility for *H* perpendicular to *b* axis is almost twenty times than that of *H* parallel to *b* axis at 2 K, which is unusual for an antiferromagnet. From the previous study^9^, the spin canting is proposed from the spectral analysis. Such specific magnetic moments configurations can account for the measured strong anisotropic susceptibility.

It is well known that the Bonner–Fisher model^8^ can describe the *S* = 1/2 uniform HAFC. There is a good initial test of whether a particular compound might be an *S* = 1/2 uniform HAFC through ${\chi }^{\mathrm{m}\mathrm{a}\mathrm{x}}\cdot {T}^{\mathrm{m}\mathrm{a}\mathrm{x}}=0.09416\frac{N{g}^{2}{{u}_{\mathrm{B}}}^{2}}{{k}_{\mathrm{B}}}$^10^, where *N* is Avogadro’s number, *g* is the spectroscopic splitting factor (*g*-factor), *µ*_*B*_ is the Bohr magneton, *k*_B_ is Boltzmann’s constant. For MACuF, we have *T*^max^ = 46.6 K and *χ*^max^ = 0.00345 emu/mol·Oe, respectively. Taking *g*_*0*_ = 2.096 from the ESR measurement at 20 K (shown as the inset of Fig. 2), we get ${\chi }^{\mathrm{m}\mathrm{a}\mathrm{x}}\cdot {T}^{\mathrm{m}\mathrm{a}\mathrm{x}}=0.09759\frac{N{g}^{2}{{u}_{\mathrm{B}}}^{2}}{{k}_{\mathrm{B}}}$. This indicates that MACuF is probably to be an uniform HAFC compound.

Generally, there is a tiny amount of paramagnetic impurities or defects in the quasi-1D materials, resulting in a Curie–Weiss term^11,12^. For example, it is found that there is ~ 2.8% of the defects of Cu^2+^ ions in BaCu_2_V_2_O_8_, contributing to the Curie tail in the susceptibility^13^. Accordingly, we fitted the susceptibility data above 4 K with the equations including a temperature independent contribution, a Curie–Weiss paramagnetic term and a term from the *S* = 1/2 uniform HAFC. Here, we assume that the weight of the contribution from paramagnetic term is *p*. So we have:

1 $$\chi \left(T\right)={\chi }_{0}+p\cdot \frac{C}{\left(T-\Theta \right)}+\left(1-p\right)\cdot {\chi }_{\mathrm{c}\mathrm{h}\mathrm{a}\mathrm{i}\mathrm{n}}(T)$$

2 $${\chi }_{\mathrm{c}\mathrm{h}\mathrm{a}\mathrm{i}\mathrm{n}}\left(T\right)=\frac{N{g}^{2}{u}_{\mathrm{B}}^{2}}{{k}_{\mathrm{B}}T}\frac{0.25+0.14995x+0.30094{x}^{2}}{1+1.9862x+0.68854{x}^{2}+6.0626{x}^{3}}$$

here, *χ*_chain_(*T*) is the Bonner–Fisher curve^14^ with *x*≡*J*/(2*k*_B_*T*), *C* is the Curie constant, and $\Theta$ is the impurity Weiss temperature. The fitted parameters are *χ*_0_ = 3.937(4) × 10^–5^ emu/mol·Oe, *p* = 1.12 × 10^–2^, *C* = 0.208 emu·K/mol·Oe, $\Theta$=3.796(7) K, *g* = 2.108(8), *J*/*k*_B_ = 72.2(1) K. It is noted the obtained *g* is very close to the value from our ESR measurement (2.096), which indicates the fitting is reasonable. The fitting results are shown in Fig. 2. The results indicate that MACuF is an ideal *S* = 1/2 HAFC system. However, in comparison with BaCu_2_V_2_O_8_, the situation is remarkably different in quite low temperature regime, where a ferromagnetic order seems to emerge upon further cooling. Such ferromagnetic order is apparently supported by the saturation of the susceptibility when the temperature approaches zero at both two direction, as shown in Fig. 2.

**Figure 3:**
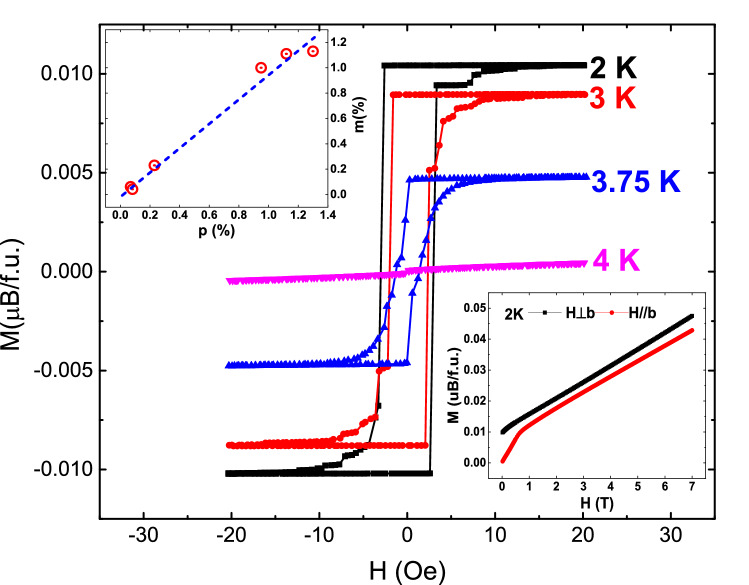
Magnetization (*M*) versus applied field (*H*) at four different temperatures for *H*⊥*b*. Top inset: paramagnetic term ratio (*p*) of six different samples obtained by fitting Eq. (2) versus the ratio of the ferromagnetic saturation magnetic moment to that of Cu^2+^(*m* = ${M}_{S}$/${M}_{{Cu}^{2+}}$). The dotted line represents the linear fitting to the data. Bottom inset: *M*–*H* curve at 2 K for *H*⊥*b* and *H*//b directions.

**Figure 4:**
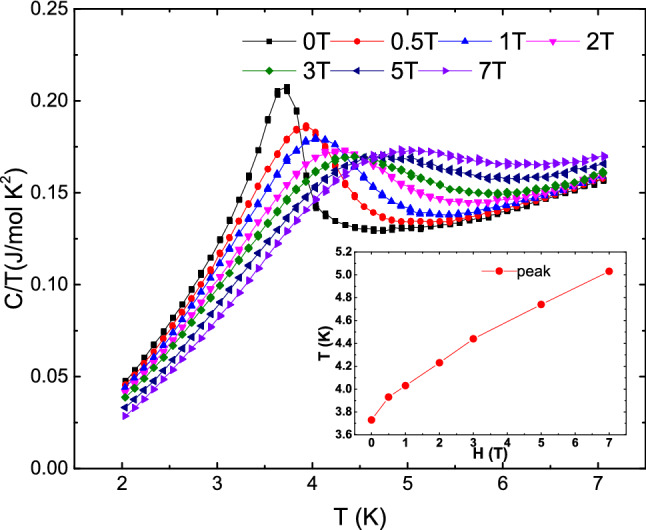
Low temperature specific heat data measured in different fields for *H*//*b* direction. The inset shows the evolution of the specific heat peak with the applied magnetic field.

To further prove the emergence of a true ferromagnetic order, we plot several isothermal *M* versus *H* curves for *H*⊥*b* in Fig. 3. One can find that *M*–*H* curves show the hysteresis loop structure when temperature is below 4 K, and the hysteresis loop disappears for *T* = 4 K. This is a strong evidence to support the ferromagnetic order below 4 K. More interestingly, it is noted that the maximum saturated magnetic moment in the ordered state is 0.0111 *µ*_B_/f.u. from Fig. 3. Comparing with the saturation moment of Cu^2+^, the ratio is *m* = ${M}_{S}$*/*${M}_{{Cu}^{2+}}$=1.06%, which is surprisingly close to the fitted paramagnetic defects concentration (*p* = 1.12%) in Eq. (1). In order to further confirm this uncommon result, several different samples were measured and analyzed by the same process. Their paramagnetic ratio (*p*) obtained by fitting Eq. (1) was basically in agreement with *m* as plotted in the top inset of Fig. 3. This proves that the ferromagnetism in MACuF is related to defects but unlikely to be originated from canted antiferromagnetic order or other aspects. The bottom inset of Fig. 3 depicts the magnetization *M* versus *H* measured at 2 K. For *H*∥*b*, *M* gradually increases from zero and increases linearly after 0.7 T. For *H*⊥*b*, a hysteresis loop is observed with the saturation magnetic field of 5 Oe and the saturation magnetic moment of *M*_S_ = 0.0111 *µ*_B_/f.u. as shown by the main plotting in Fig. 3. After deducting the constant term and the contribution of HAFC, *M* increases at the same rate as for *H*∥*b* after 0.7 T. This is similar with the magnetization of hard and easy axis of a ferromagnet. The different behaviors of *M*–*H* at 2 K for two different directions are consistent with the strong anisotropic susceptibilities shown in Fig. 1, and can be also understood by the spin canting effect. The long-range ferromagnetic phase transition at 4 K is settled down by the specific heat measurement, as shown in Fig. 4. There is a *λ*-like peak in the specific heat curve, and the anomalous peak moves toward high temperature as external magnetic field increases. These are the typical behaviors of ferromagnetic phase transition under magnetic fields. We also measured the zero field specific heat of the sample used in Fig. 2 labeled as sample 1 shown in Supplementary Information. It is found the magnetic entropy integrated from the $\lambda$-like peak also shows a qualitative match with the Cu^2+^ defect concentration.

It is easy to understand that some broken chains, including some surfaces of the crystal, will inevitably appear during the growth of quasi-1D chain crystals. These broken chains may exist in the form of isolated point defects or localized surface defects inside the crystal. The magnetic moments existing as surface defects could be considered as independent systems and can form long-range ordered states. For MACuF single crystal, it is reasonable to believe that the long-range ferromagnetic order observed at 4 K is attributed to the Cu^2+^ ions from localized surface defects in the 2D plane, *i.e.*, the *ac* plane, as shown in Fig. 1b. This situation is similar to the ferromagnetism observed in Teflon tape^15^. When a Teflon tape is cut or stretched, there will be many carbon dangling bonds, and each of them carries a magnetic moment of 1 *µ*_B_. The moments could show ferromagnetic coupling when carbon dangling bonds formed a 2D network^1,5^.

Such an unexpected long-range ferromagnetic order observed in MACuF is distinctly different from many other HAFC materials, where the interchain coupling usually results in the long-range antiferromagnetic order with very different ordering temperature^16–26^. Here, we provide the solid evidence to prove the long-range ferromagnetic order can be attributed to the Cu^2+^ endpoint of the chains, highlighting the important role of the broken chains. Our studies provide a valuable view to help to understand the phase transitions in HAFCs.

## Conclusion

The magnetic susceptibility and specific heat data of single-crystal MACuF have been measured. The susceptibility above 4 K was decomposed using a Curie–Weiss paramagnetic term and the *S* = 1/2 uniform 1D HAFC model. Combining with specific heat results under magnetic fields, we prove the magnetic phase transition at 4 K to the formation of long-range ferromagnetic order arising from Cu^2+^ in the 2D plane constructed by endpoints of the chains.

## Method

Single crystals of MACuF with dimensions up to 3 × 3 × 0.5 mm^3^ were prepared by solvothermal method following Ref.^7^. Different evaporation rate is used to obtain samples with different defects concentration. Static magnetic properties were measured with a Quantum Design MPMS-3 superconducting quantum interference device magnetometer between 2 and 300 K in an applied field up to 7 T. The sensitivity of SQUID is ${10}^{-8}$ emu, which satisfies the measurement of very small magnetic moment in our study. The ESR measurements were performed in a Bruker EMXplus10/12 CW spectrometer at X-band frequencies equipped with a continuous He-gas flow cryostat (Oxford Instruments) working in the temperature range 1.8 ≤ *T* ≤ 300 K. Specific heat was measured by a pulse relaxation method using a commercial calorimeter (Quantum Design PPMS).

## Supplementary Information

Supplementary Figures.
