# Supplementary material for: Defect-induced ferromagnetism in a S = 1/2 quasi-one-dimensional Heisenberg antiferromagnetic chain compound
Source: Sci Rep. 2021 Jul 14;11:14442. doi: 10.1038/s41598-021-93930-1 (PMC8280101; doi:10.1038/s41598-021-93930-1)
Supplement: Supplementary file 1 — Supplementary Figures. [file 41598_2021_93930_MOESM1_ESM.pdf]

Defect-induced ferromagnetism in a S=1/2 quasi-one-dimensional Heisenberg antiferromagnetic chain compound

Zhe Wang,[1,2] Lin Hu,[1,3] Langsheng Lin[1], Yuyan Han[1], Ning Hao,[1] Jingtao Xu,[4] Qianwang Chen,[1,5] and Zhe Qu[1,3]

[1] *Anhui Key Laboratory of Condensed Matter Physics at Extreme Conditions, High Magnetic Field Laboratory, Hefei Institutes of Physical Sciences, Chinese Academy of Sciences, Hefei, Anhui 230031, China.*

[2] *Science Island Branch of Graduate School, University of Science and Technology of China, Hefei, Anhui 230026, China.*

[3]*CAS Key Lab of Photovoltaic and Energy Conservation Materials, Hefei Institutes of Physical Sciences, Chinese Academy of Sciences, Hefei, Anhui 230031, China.*

[4] *Ningbo Ruiling Advanced Energy Materials Institute Co., Ltd, Ningbo, Zhejiang 315500, China*

[5] *Hefei National Laboratory for Physical Sciences at Microscale, Department of Materials Science & Engineering, University of Science and Technology of China, Hefei, 230026, Anhui, China*

Supplementary Information

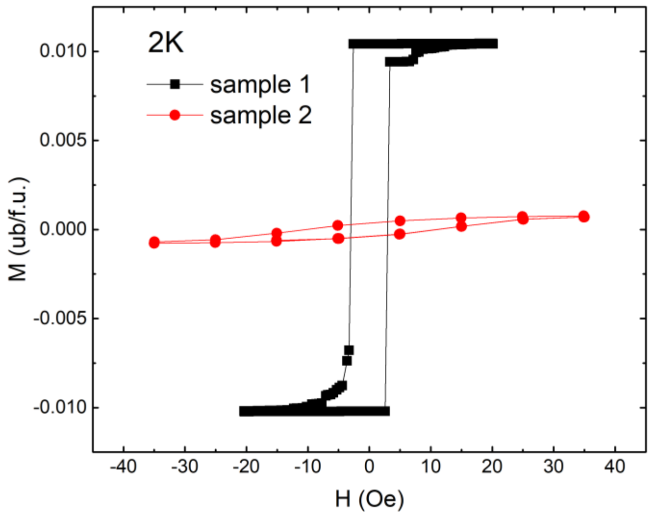

Supplementary Figure S1 – MH profiles at 2 K for sample 1 and sample 2. Note the huge difference in saturating field.

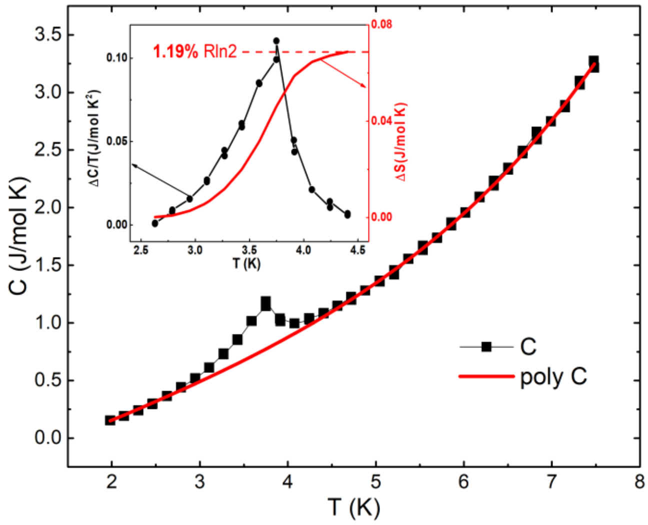

Supplementary Figure S2– Heat capacity of sample 1, poly C is a polynomial background fitting to extract the specific heat of the FM phase transition. Inset represent the change of specific heat and entropy caused by the FM phase transition showing good consistency with its defect concentration(p=1.12%).
